# ChIP provides 10-fold microbial DNA enrichment from tissue while minimizing bias

**DOI:** 10.1007/s11033-025-10330-8

**Published:** 2025-02-21

**Authors:** Shrikant Bhute, Jon G. Sanders, Se Jin Song, Sydney Lavoie, Austin Swafford, Caitlin Guccione, Lucas Patel, Antonio Gonzalez, Michelle G. Rooks, Rob Knight, Andrew Bartko

**Affiliations:** 1 Center for Microbiome Innovation, University of California San Diego, La Jolla, San Diego, CA USA; 2 Pfizer Research and Development, Cambridge, MA USA; 3 Department of Pediatrics, University of California San Diego, La Jolla, San Diego, CA USA; 4 Bioinformatics and Systems Biology Program, University of California San Diego, La Jolla, San Diego, CA USA; 5 Medical Scientist Training Program, University of California San Diego, La Jolla, San Diego, CA USA; 6 Department of Computer Science and Engineering, University of California San Diego, La Jolla, San Diego, CA USA; 7 Department of Bioengineering, University of California San Diego, La Jolla, San Diego, CA USA

**Keywords:** Microbiome, Host depletion, Frozen tissue, Chromatin immunoprecipitation

## Abstract

**Background:**

Host DNA depletion is a critical tool for accessing the microbiomes of samples that have a small amount of microbial DNA contained in a high host background. Of critical practical importance is the ability to identify microbial DNA sequences in frozen tissue specimens. Here, we compare four existing commercial methods and two newly introduced methods involving chromatin immunoprecipitation (ChIP) on frozen human and pig intestinal biopsies.

**Results:**

We find that all methods that rely on differential lysis of host and microbial cells introduce substantial biases as assessed by 16 S rRNA gene amplicon profiling. However, ChIP enables 10-fold enrichment of microbial DNA while introducing less bias, sufficient to make assessment possible against background, in both pigs and humans.

**Conclusions:**

We recommend ChIP in situations where host depletion is important but where minimizing taxonomic bias is essential, and the MolYsis or Zymo kit for situations where host depletion level is more important than taxonomic bias.

**Conclusions:**

We recommend ChIP in situations where host depletion is important but where minimizing taxonomic bias is essential, and the MolYsis or Zymo kit for situations where host depletion level is more important than taxonomic bias.

**Supplementary Information:**

The online version contains supplementary material available at 10.1007/s11033-025-10330-8.

## Importance

Many samples that would be interesting to examine for microbes consist mostly of host cells and host DNA. Comparisons of methods to address this problem have mostly focused on the amount of host DNA removed, not whether the same microbes are identified in the samples before and after removal of host DNA. In this paper, we compare four different commercially available methods based on different principles, and also introduce two new methods based on a new principle for separating bacterial and host DNA. We show that one of the new methods is able to enrich microbial DNA with minimal bias even in a high host background. However, we do note that some of the commercial methods are able to remove the host DNA much more efficiently, although at the cost of introducing more bias. This study is important because identifying microbes in tissues ranging from lungs to gastrointestinal samples is of increasing interest.

## Background

Metagenomic sequencing potentially offers less bias and broader taxonomic coverage than 16S rRNA gene amplicon sequencing (16S), while also allowing characterization of the full gene repertoire and accessing fungi and viruses. However, host tissue samples require cost-prohibitive depth of sequencing for sufficient microbial coverage. At the threshold of 500,000 microbial reads required for shallow-coverage metagenomics with SHOGUN [1], for example, a specimen yielding 0.1% microbial reads would require 50 million total reads for analysis.

Host depletion methods, which reduce the amount of host DNA prior to sequencing, have been demonstrated for a range of biospecimen types [2–4], including commercial protocols, which have recently been compared for vaginal, skin, and saliva samples [5].

Commercial host depletion kits include MolYsis Basic5 (MOL), QIAamp DNA Microbiome Kit (QIA), HostZERO Microbial DNA Kit (ZYM), and NEBNext Microbiome DNA Enrichment Kit (NEB). Three of these kits (MOL, QIA, and ZYM) target host DNA by degrading extracellular nucleic acids based on three principles: (1) differential lysis of host cells, (2) centrifugal enrichment of unlysed microbial cells, and (3) degradation of accessible, cell-free nucleic acids. While preservation of samples with glycerol can help protect microbial cells from lysis during storage to support host-depletion using these principles [2], for many banked and archival samples, either no preservative or another preservation media may have been used that disrupts microbial membranes, leaving microbial DNA susceptible to degradation. Therefore, these approaches could introduce bias at each step if different bacteria are differentially affected by the storage process. In contrast, NEB focuses on removing host DNA by affinity-based separation of methylated CpG sequences. This protocol is based on three assumptions: (1) host DNA is accessible in solution, (2) methylated CpG is only found on host DNA and is present and available for binding on all contaminating host DNA fragments, and (3) magnetic beads successfully pull targeted material out of solution. While cell lysis and magnetic bead isolation are robust technologies, highly fragmented host DNA may not contain sufficient methylated CpG fragments to be isolated and CpG elements may be present in bacterial DNA [6], as well as in fungi.

Although substantial progress has been made in host DNA depletion from liquid specimens such as saliva [2] and fresh tissue [4], the key problem of depleting host DNA from frozen bulk tissue samples has been inadequately addressed and has typically resulted in low levels of enrichment using existing methods [7]. Here, we address this problem in intestinal biopsies from humans and pigs. We compare the four above commercial methods to two new methods we developed based on chromatin immunoprecipitation (ChIP), and assess both host yield and taxonomic bias.

ChIP binds and removes histone-bound host DNA using antibodies coupled to magnetic beads. This approach does not rely on intact microbial cells, and unlike other commercial methods, cell-free microbial DNA should not be affected. Our modified-ChIP protocol (mChIP) adds 10 min of 15,000 xg centrifugation prior to immunoprecipitation as a physical separation step. Centrifugal separation is the only variable that differs between ChIP and mChIP, allowing evaluation of the impact of this variable.

**Fig. 1:**
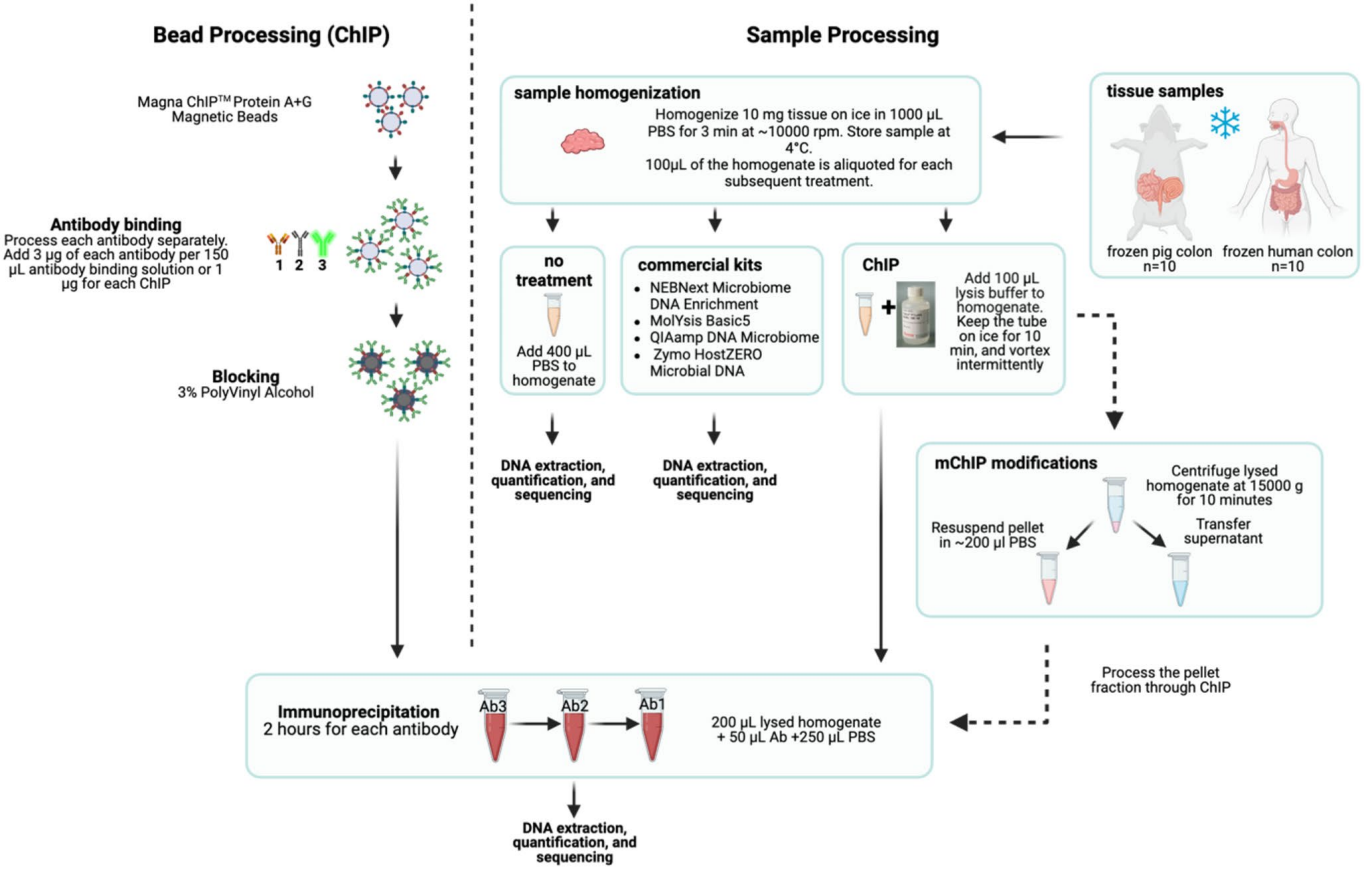
Study overview. Frozen colon tissue samples were sourced from 10 human individuals and 10 Yucatan pigs. Homogenization of tissue was performed prior to running host depletion protocols. Protocols for the commercially available kits followed manufacturers’ instructions. The protocol design for ChIP with respective steps for antibody bead processing is shown to the left of the dashed line while sample processing is shown to the right of the dashed line. The modification for the mChIP protocol is shown in a separate box. Processed beads and samples were combined stepwise to allow immunoprecipitation of host DNA using each of the 3 antibody-bound beads before undergoing DNA extraction, quantification, and sequencing

Our study design (Fig. 1; see also Methods) was as follows. We collected colon biopsies from 10 human subjects and 10 Yucatan pigs. Human samples were mucosal biopsies taken during colonoscopies, while pig samples were sections of the entire gastrointestinal tube excised from euthanized subjects. Existing commercial protocols do not specify how to process biopsies, so we used the *Qiagen TissueRuptorII* to mechanically disrupt the extracellular matrix [8]. 100 µL aliquots (containing ∼ 1 mg tissue) of each sample were used in the six host depletion methods (MOL, QIA, ZYM, NEM, ChIP, and mChIP) plus non-depleted control samples (Table S1). We then performed 16S and shotgun metagenomics to assess taxonomic bias and level of host depletion, respectively.

## Results

**Fig. 2:**
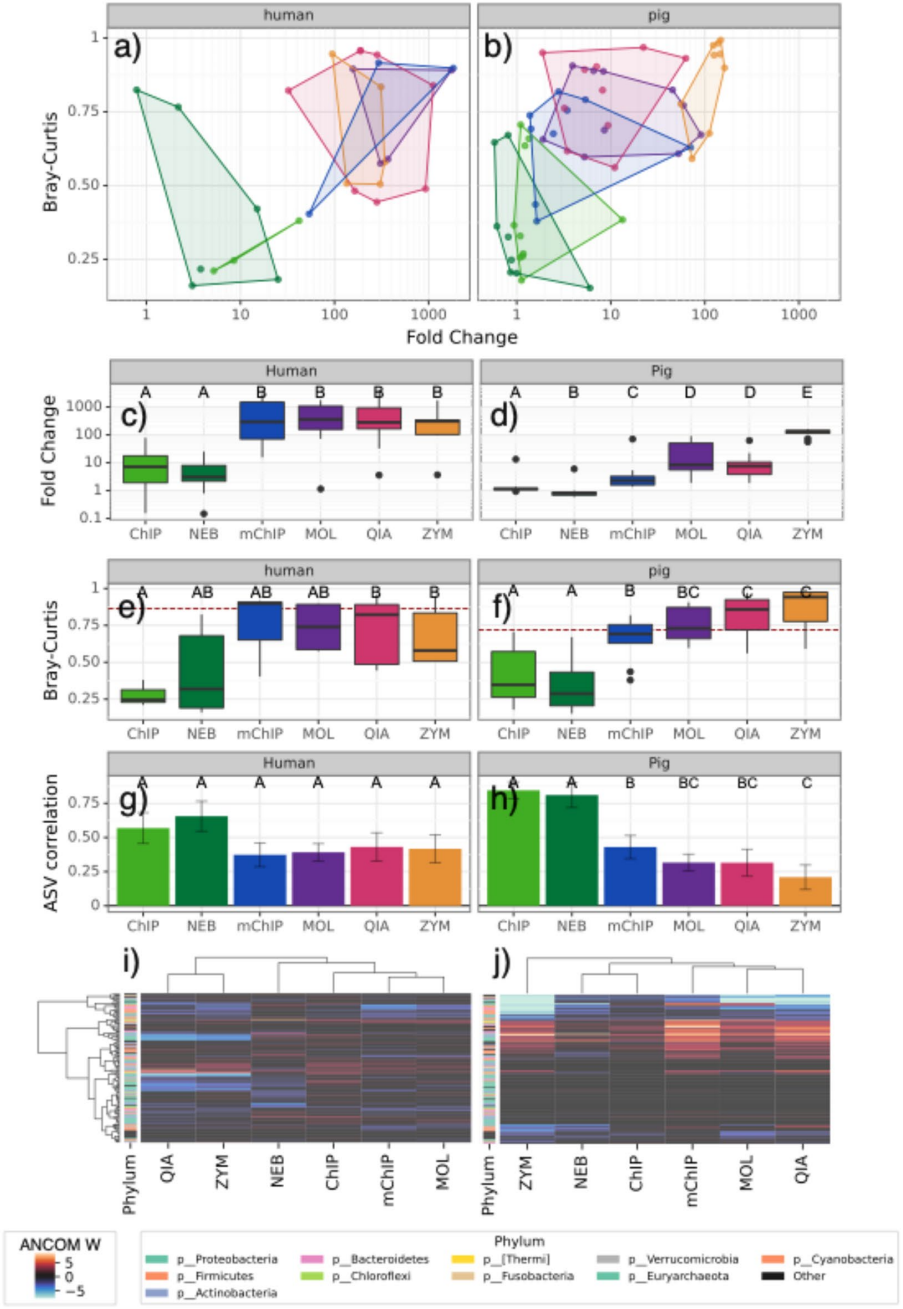
Trade-off between microbial enrichment and bias when comparing samples depleted using each of the described methods to the non-depleted sample for each subject. 2**a**: Fold change in microbial DNA percentage obtained using shotgun metagenomics (x axis) versus difference from non-depleted 16 S rRNA results via Bray-Curtis (y axis) in human samples. Different colors show the convex hull of points obtained for samples using different depletion methods (ChIP: light green; NEB: dark green; mChIP: blue; MOL: purple; QIA: pink; ZYM: orange). 2**b**: As for 2a, but for pig samples. 2**c**: Distribution of fold-change results for each method, shown as box-and-whisker plots. Comparisons that are significantly different (*p* < 0.05) based on a Mann-Whitney U test are denoted by different letters above the box-and-whisker plots. 2**d**: As for 2**c**, but for pig samples. 2**e**: Bray-Curtis dissimilarity between no depletion and each method for each human sample, shown as distribution of dissimilarities for each method. Comparisons that are significantly different (*p* < 0.05) based on a Mann-Whitney U test are denoted by different letters above the box-and-whisker plots. 2**f**: As for 2**d**, but for pig samples. 2**g**: Distribution of correlations between ASV (Amplicon Sequence Variant) abundance in non-depleted versus depleted samples using Spearman’s rho, for human samples. Comparisons that are significantly different (*p* < 0.05) based on a Mann-Whitney U test are denoted by different letters above the box-and-whisker plots. 2**h**: As for 2**f**, but for pig samples. 2**i**: Heat map showing ASVs collapsed at the family level, assessed using ANCOM-BC as differentially abundant between paired depleted and non-depleted samples using at least one of the methods (W value indicated by the color). 2j: As for 2i, but with pig samples

The assessed methods differed markedly both with respect to microbial enrichment and bias (Fig. 2; Supplementary Material 2 Tables S3–S6), with enrichment generally being greater for human than pig samples (Fig. 2a-d; Supplementary Material 3) and bias comparable between the two species (Fig. 2a-b, e-h). This difference in performance between the host types may be due to the GI sections potentially having a higher relative proportion of host cells to microbial cells than the mucosal layer from the human samples. In human samples, ChIP yielded ∼ 10-fold enrichment, NEB ∼ 5-fold, and other strategies 100-fold or greater (Fig. 2c; Supplementary Material 2 Table S3). In pig samples, performance was substantially worse, with NEB and ChIP showing the lowest enrichment, typically barely above 1 for ChIP and often *below* 1 for NEB, with ZYM yielding > 100-fold enrichment and other methods performing at intermediate levels (Fig. 2d; Supplementary Table 2 Table S4). Of more concern, the recovered microbial communities after depletion were radically different from the original communities as assessed by 16S (Supplementary Material 2 Tables S3–S4; Kruskal-Wallis test H-statistic = 10.914, p-value = 5.312e-02 in humans; H-statistic = 46.340; p-value = 7.743e-09 in pigs). ChIP yielded low mean Bray-Curtis distances relative to non-depleted controls of ∼ 0.25 for humans and ∼ 0.3 for pigs, with relatively low variation in humans and higher variation in pigs; while NEB yielded comparable means but much higher variation in humans specifically. However, all other methods yielded extremely dissimilar communities, often in the range of 0.8 or above (Fig. 2e-f). Consistent with these observations, the correlations between ASV (Amplicon Sequence Variant) abundances in non-depleted samples and depleted samples trended higher for ChIP and NEB than for the other methods, averaging ∼ 0.5 and ∼ 0.6 in humans, respectively, and ∼ 0.8 and ∼ 0.75 in pigs, respectively; whereas the other methods averaged around ∼ 0.3 (Fig. 2g-h; Tables S3-S4). An analysis using ANCOM-BC [9] to identify taxa that are differentially abundant between paired depleted and non-depleted samples indicate that while some of the most highly affected taxa are consistent across the methods depending on their mechanism of action, many differ across the methods and in particular, across the host types (Fig. 2i-j; Supplementary Material 2 Tables S5-S6).

Of the two depletion methods that rely solely on host DNA pulldown, ChIP trended towards higher fold change in microbial enrichment in both humans and pigs (Fig. 2c-d). In contrast, depletion methods that use physical separation and DNA degradation had much higher fold change in microbial enrichment than protocols that instead rely on host DNA pulldown. However, these methods also introduced much greater biases, as measured by Bray-Curtis (Fig. 2e-f) (we obtained similar results with the Jaccard index, which is a qualitative rather than quantitative method; data not shown).

## Discussion

An important practical consideration is the ability to process frozen specimens. All specimens in this study were frozen prior to processing. MOL, QIA, and ZYM rely on the degradation of extracellular DNA, and microbial cells may not remain intact when frozen without a preservative. CHIP and NEB do not suffer from this limitation because they rely on separation of host from non-host DNA directly, which may explain their improved performance for retaining overall community similarity to the non-depleted sample. Host-replete sample types where extracellular microbial DNA is of potential interest, such as mammalian blood, may also benefit from these methods. Future work comparing frozen to non-frozen tissue would be valuable for resolving that question, although this was beyond the scope of our present work focusing on methods for frozen specimens.

## Conclusions

We conclude that ChIP provides ∼ 10-fold microbial enrichment while maintaining low taxonomic bias. We also conclude that MOL and ZYM provide the best depletion overall, albeit at the cost of high bias, and should be used in discovery settings where the ability to detect some microbes outweighs the drawback that many are missed.

## Methods

### Human colon specimen collection

Ten human colon samples were procured from UCSD’s Moores Cancer Center biorepository. Samples were flash-frozen in liquid nitrogen and stored at -80 °C for 48 h before homogenization.

### Pig colon specimen collection

All procedures performed on animals were in accordance with regulations and established guidelines and were reviewed and approved by UC San Diego’s Institutional Animal Care and Use Committee (protocol S21167). Ten Yucatan pigs were maintained at UCSD’s Elliott Field Station. Throughout the study, the pigs were fed with an 80:20 mix of LabDiets’s 5L80 and 5084 diets. Of the ten pigs, five pigs were given amoxicillin (20 mg/Kg body weight) for 10 days, and five were not given antibiotics. Three months post antibiotic treatment, when the pigs were ∼ 6 months old, they were euthanized and their GI tracts were harvested. Colon tissues were chopped into small pieces and placed in Eppendorf tubes. Collection tubes were kept on ice during the sampling, and then transferred to a -20 °C freezer. Finally, samples were transferred to a -80 °C freezer for long-term storage until further processing.

### Homogenization of the tissue samples

Tissue homogenization was performed in the biosafety cabinet. Frozen human (*n* = 10) and pig (*n* = 10) colon samples (rectum samples stored without preservatives) were thawed on ice for 5–10 min. Approximately 10 mg tissue chucks were placed in a sterile 2 mL Eppendorf tube and homogenized in 800 µL of sterile PBS using a handheld motorized homogenizer (Qiagen TissueRuptor II). The tubes were kept on ice during homogenization, and homogenization was performed by immersing the probe in the lysis buffer without any beads at a speed of 10,000 rpm for three minutes. During this step, the homogenization probe was moved up and down a few times to ensure complete tissue homogenization. At the end of homogenization, a total of 200 µL of PBS was added over the inner and outer walls of the probes to collect the adhering tissue homogenate as much as possible. The resulting tissue homogenate was centrifuged briefly to collect homogenate at the bottom and kept on ice or at 4 °C. From this source tube, 100 µL of tissue was used for each of the host depletion methods described below.

### Host depletion using chromatin immunoprecipitation (ChIP)

10 µL of Magna ChIP Protein A + G Magnetic Beads were mixed with 190 µL Bead Wash Solution (1X PBS containing 1X HALT Protease Inhibitor and 0.05% Tween 20). Beads were collected using a magnetic stand, and the supernatant was discarded. Beads were then washed twice with 500 µL Bead Wash Solution and resuspended in 50 µL of Bead Wash Solution. Antibodies were then allowed to bind to the protein A + G beads. Fifty µL of washed beads were added to the 500 µL bead wash solution, and 3 µL of antibody was added in a separate tube. The three antibodies were (1) Anti-DNA/Histone H1 Antibody, (2) Anti-Histone H4 Antibody, pan, clone 62–10 C-2 (rabbit monoclonal), and (3) Anti-Dimethyl-Histone H3 (Lys4) Antibody clone CMA303 (mouse monoclonal), all manufactured by EMD Millipore. Tubes were then rotated at 4 ℃ for one hour at the speed of 30 RPM, centrifuged to collect the beads, placed on a magnetic stand, and the supernatant was discarded. Without removing the tubes from the magnet, 200 µL of 3% PVA (Poly-vinyl-alcohol) was added to the tubes, and beads were resuspended by vortexing the tubes gently. Tubes were then rotated at 4 ℃ for 30 min at the speed of 30 RPM, centrifuged to collect the beads, placed on a magnetic stand, and removed the supernatant. Finally, antibody-immobilized and PVA-blocked beads were washed with 200 µL Bead Wash Solution and resuspended in 150 µL of Bead Wash Solution. The number of tubes needed depended on the number of samples to be processed. One tube of 150 µL antibody was sufficient for 3 samples.

100 µL tissue homogenate (both human and pig) was lysed with 100 µL Pierce Lysis buffer containing 1X HALT Protease Inhibitor. The lysis was performed on ice for 10 min with periodic vortexing. The resulting tissue lysate was subjected to sequential chromatin immunoprecipitation (ChIP) by adding 200 µL tissue lysate, 50 µL of Anti-Dimethyl-Histone H3 bound protein A + G beads (Ab3), and 250 µL of Bead Wash Solution. The mixture was rotated at 4 ℃ for two hours at the speed of 10 RPM. After two hours, tubes were centrifuged briefly to collect the beads and placed on a magnetic stand to collect the chromatin-bound beads. Supernatant was collected in a new tube, and 50 µL Anti-Histone H4 Antibody, pan, clone 62–10 C-2 bound beads (Ab2) were added to the supernatant. The resulting mixture was then rotated at 4 ℃ for two hours at the speed of 10 RPM. Finally, the process was repeated for Anti-DNA/Histone H1 Antibody (Ab1). After the final round of ChIP, the supernatant was collected in a fresh tube and used for DNA extraction.

### Modification of ChIP (mChIP)

100 µL of tissue homogenate was lysed with 100 µL Pierce Lysis buffer containing 1X HALT Protease Inhibitor. The lysis was performed on ice for 10 min with periodic vortexing. This mixture was then centrifuged at 15,000xg for 10 min to pellet intact cells along with heavier debris. The supernatant was transferred out of the tube, and the pellet was resuspended in 200 µL PBS before undergoing sequential chromatin immunoprecipitation as described above.

### Host depletion using MolYsis Basic5 (MOL)

For this method, the sample volume was adjusted to 1 mL using the buffer SU provided in the kit, and the depletion was performed as described in the user manual. Briefly, samples were treated with 250 µl buffer CM for 5 min, followed by buffer DB1 (250 µl) and MolDNase B (10 µl) treatment for 15 min. The tubes were then centrifuged at 12,000xg for 10 min, and the pellet was resuspended in 1 ml buffer RS and centrifuged again at 12,000xg for 5 min to remove residual MolDNase B activity. Microbial cells were then lysed using 80 µl buffer RL, 20 µl BugLysis solution, and 1.4 µl β-mercaptoethanol. The DNA was extracted using MagMAX™ Microbiome Ultra Nucleic Acid Isolation Kit, which also included proteinase K treatment, and the DNA was eluted in 50 µL of Tris-HCl (pH 8). The resulting DNA was stored at − 20 °C until further processing.

### Host depletion using QIAamp DNA microbiome kit (QIA)

For each human and pig colon homogenate, the sample volume was adjusted with sterile PBS to the kit’s recommended volume of 1 mL, and the samples were processed as per the manufacturer’s instruction. Briefly, in a 2 mL Eppendorf tube, 500 µL of Buffer AHL was added to a 1 mL sample, followed by 30 min of room temperature incubation. Samples were then centrifuged at 10,000 xg for 10 min, and the supernatant was discarded. A mixture of 190 µL Buffer RDD and 2.5 µL Benzonase was added to each tube and incubated at 37 °C for 30 min on an Eppendorf thermomixer with shaking at 600 RPM. 20 µL Proteinase K was added to each sample and incubated at 56 °C for 30 min on an Eppendorf thermomixer with shaking at 600 RPM. Next, 200 µL Buffer ATL was added to each sample, and all content was transferred into Pathogen Lysis Tube L. Microbial lysis was performed using Fisherbrand Beadmeal at the speed of 6.5 m/s in two rounds, each one for 45s with a five-minute interval. Pathogen Lysis Tube L was then centrifuged at 10,000 xg for 1 min, and the content was transferred to a fresh microcentrifuge tube. Samples were treated with 40 µL Proteinase K for 30 min at 56 °C followed by 200 µL Buffer APL treatment for 10 min at 70 °C. Finally, 200 µL ethanol was added to the lysate, and ∼ 700 µL of the mixture was transferred to the QIAamp UCP Mini spin column and centrifuged at 6,000 x g for 1 min. The columns were washed with 500 µL of each Buffer AW1 and AW2. After the final centrifugation of the washing steps, 50 µL buffer AVE was applied, the columns were incubated for 5 min at RT, and the DNA was eluted by centrifuging at 10,000 xg for 1 min. The final DNA was stored at − 20 °C until further processing.

### Host depletion using HostZERO microbial DNA kit (ZYM)

For this method, sample volume was adjusted to 200 µL (100 µL tissue homogenate and 100 µL sterile PBS) to match the kit recommended sample volume and the samples were processed per the manufacturer’s instructions. Briefly, 1mL of Host Depletion Solution was added to each sample, and the host lysis was performed for 15 min by end-over-end rotation at room temperature. Tubes were then centrifuged at 10,000 x g for 5 min, and the supernatant was discarded. The pellet was resuspended in 100 µL, and host DNA depletion was performed by adding 1 µL of Microbial Selection Enzyme at 37 °C for 30 min. Samples were then treated with 20 µL of Proteinase K and incubated at 55 °C for 10 min, and 100 µL of 2X concentrate of DNA/RNA Shield was added. Entire samples were transferred to ZR BashingBead™ Lysis Tube containing 750 µL of ZymoBIOMICS Lysis Solution and lysed using Fisherbrand Beadmeal at 5 m/s speed for 5 min. Next, 400 µL of supernatant was transferred to a collection tube and treated with 1200 µL ZymoBIOMICS DNA Binding Buffer. The resulting mixture was passed through the Zymo-SpinTM IC-Z Column and washed once with 400 µL of ZymoBIOMICS DNA Wash Buffer 1 and twice with Wash Buffer 2 (700 µL and 200 µL each) in a sequential manner. After the final centrifugation of the washing steps, 50 µL DNase-free water was applied, the columns were incubated for 5 min, and DNA was eluted by centrifugation at 10,000 xg for 1 min. The resulting DNA was stored at − 20 °C until further processing.

### Host depletion using NEBNext Microbiome DNA Enrichment kit (NEB)

Total DNA from 100 µL tissue homogenate was extracted using MagMAX™ Microbiome Ultra Nucleic Acid Isolation Kit and used as input for the NEBNext Microbiome DNA enrichment kit. Briefly, 160 µL of MBD2-Fc-bound protein magnetic beads were prepared according to the manufacturer’s instructions. The CpG-methylated host DNA was removed from the sample by mixing 50 µL DNA and 160 µL of MBD2-Fc-bound protein magnetic beads. During this step, the tubes were rotated for 15 min at room temperature. The MBD2-Fc-bound host DNA was then removed by placing the tubes on a magnetic rack, and the supernatant was then transferred to a fresh tube. Microbial DNA in the supernatant was then concentrated using 1.8X volume of Quntabio sparQ PureMag Beads, washed twice with 80% ethanol, and eluted in 50 µL of Tris-HCl (pH 8). The resulting DNA was stored at − 20 °C until further processing.

### DNA extraction and quantification

The DNA from control samples (non-host depleted samples), ChIP samples, and MolYsis samples were extracted using the MagMAX™ Microbiome Ultra Nucleic Acid Isolation Kit following the manufacturer’s instructions. The microbial cells were lysed using Fisherbrand Beadmeal for 5 min at the speed of 5 m/s and processed further on KingFisher automated DNA extraction and purification system. DNA quantification was performed using Qubit dsDNA HS Assay Kit.

### Sequencing

The V4 region of the 16S rRNA gene was amplified using primers with unique forward primer barcodes (515fB-806r), following the Earth Microbiome Project protocol [10], and sequenced on the Illumina MiSeq sequencing platform with paired-end 150 bp cycles.

Metagenomic libraries from control and host depleted samples were prepared using a 1:10 miniaturized version of KAPA Hyper-Plus Kit (Roche) using nanoliter-scale liquid handling robots and as per the manufacturer’s instructions. For samples with less than 1 ng DNA, a maximum volume of 3.5 µL input DNA was used. The resulting libraries were quantified using Pico Green Quantification Kit, normalized, and sequenced on the iSeq 100 system to assess the read distribution [11]. Based on the iSeq data, libraries were re-pooled to have optimal read distribution per sample, and samples were sequenced on an S4 flowcell of an Illumina NovaSeq 6000, sequenced as a paired-end 150-cycle run at the UCSD IGM Genomics Center with a median sequencing depth of a 5.8 million reads per sample.

### Bioinformatic analysis

Raw FASTQ files were quality filtered using fastp (v. 0.20.1) [12] with a minimum length cutoff of 100 base pairs and subject to adapter removal using the full list of adapters included in Supplementary Material 2 Table S2. Human host reads were removed using a three-step process: first, reads were aligned to the human reference genome GRCh38.p14 [13] using minimap2 (v. 2.28-r1209) [14] and unaligned reads were extracted using samtools (v. 1.12) [15]; next alignment was repeated using the human reference genome T2T-CHM13v2.0 [16]; finally a pangenome index was constructed from the 94 human reference genomes from the Human Pangenome Reference Consortium (HPRC) [17] using Movi [18] (unversioned; git commit hash 76d5a6da1ec0aeb0121b5ac7c59b295936e23cc1) and movi-default was used to query and discard any remaining reads with high pseudo-matching lengths. Pig host reads were removed by aligning against NCBI’s *Sus scrofa* 11.1 genome. Reads not matching the target genomes were assumed to be microbial for the purposes of this analysis.

Fold change was calculated on a per-sample basis, as the proportion of microbial reads in the depleted sample divided by the proportion of microbial reads observed in the same sample’s non-depleted control replicate. For 16 S rRNA gene sequence data, samples were processed in Qiita [19] using the deblur [20] Amplicon Sequence Variant (ASV) workflow. ASVs that were successfully inserted into the reference GreenGenes_13.8 phylogeny were retained for downstream diversity analysis using QIIME2 [21]. Bray-Curtis dissimilarity values were used to describe between-group differences using sample tables rarefied to 1000 observations per sample, and taxonomic differential abundance calculations were performed using ANCOM-BC [9] on non-rarefied GreenGenes taxonomy tables collapsed to the level of family.

## Electronic supplementary material

Below is the link to the electronic supplementary material.

Supplementary Material 1

Supplementary Material 2

Supplementary Material 3

## Data Availability

Sequence data (after removal of reads matching human reference genomes) are available in EMBL’s European Bioinformatics Institute (EBI) European Nucleotide Archive (ENA) with project accession PRJEB81804. They are also available in Qiita (https://qiita.ucsd.edu) under study 14778, prep 15337 (16S) and prep 17451 (metagenomic). The processed 16S feature table is additionally provided as a supplementary data file (Supplementary Material 1). Sample associated metadata, including sample accession numbers and metagenomic sequence counts before and after host read filtering, are available in Supplementary Material 2 Table S1.
