# Supplementary figures and images for: ChIP provides 10-fold microbial DNA enrichment from tissue while minimizing bias
Source: Mol Biol Rep. 2025 Feb 21;52(1):258. doi: 10.1007/s11033-025-10330-8 (PMC11845529; doi:10.1007/s11033-025-10330-8)

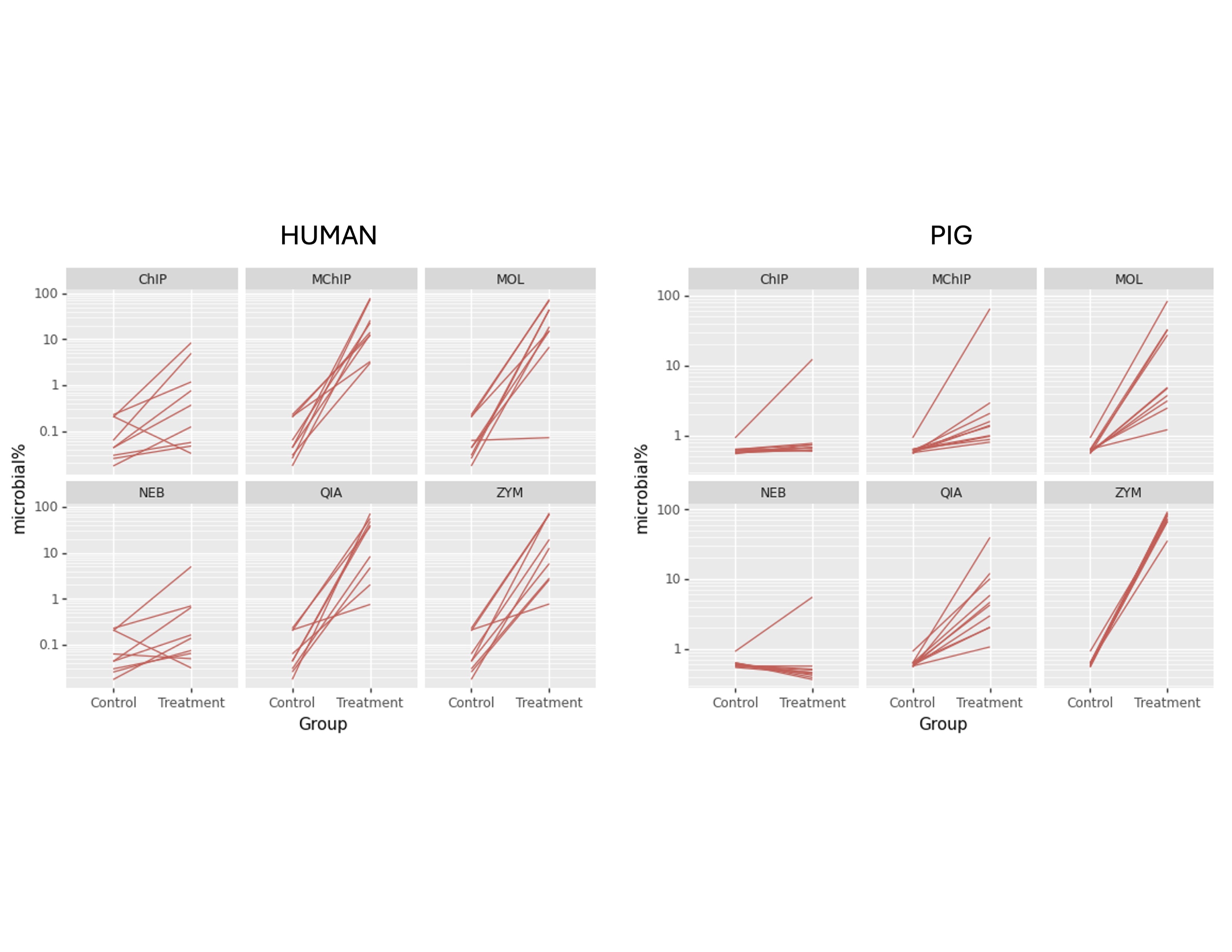

Supplement: Supplementary file 3 — Supplementary Material 3 [file 11033_2025_10330_MOESM3_ESM.jpg]
